# Relationship between body weight and daytime sleepiness in patients with untreated OSA: The role of hypoventilation due to obesity

**DOI:** 10.1007/s11325-026-03658-3

**Published:** 2026-03-22

**Authors:** Giulia Sartori, Alberto Fantin, Gianluigi Dorelli, Marcello Ferrari, Ernesto Crisafulli

**Affiliations:** 1 Department of Medicine, Respiratory Medicine Unit, University of Verona and Azienda Ospedaliera Universitaria Integrata of Verona, Largo L.A. Scuro, 10, Verona, 37134 Italy; 2 Department of Pulmonology, S. Maria della Misericordia University Hospital, Udine, Italy; 3 School of Medicine in Sports and Exercise, University of Verona, Verona, Italy

**Keywords:** Obstructive sleep apnoea, Body weight, Daytime sleepiness, Obesity-hypoventilation

## Abstract

**Purpose:**

To evaluate the relationship between body weight and daytime sleepiness (DS) in patients with obstructive sleep apnoea (OSA).

**Methods:**

We prospectively evaluated 353 untreated patients with OSA, categorised by body mass index (BMI) as normal weight, overweight, grade 1, grade 2, or grade 3 obesity. Patients with OSA and obesity hypoventilation syndrome (OHS) were analysed as a separate group. Demographic and anthropometric data, nocturnal polygraphy parameters, and DS (by the Epworth Sleepiness Scale-ESS) were collected. Excessive DS was defined as ESS > 10.

**Results:**

Patients were classified as normal weight (13%), overweight (26%), grade 1 obesity (28%), grade 2 obesity (16%), grade 3 obesity (6%), and OHS (11%). DS did not differ significantly across body weight categories, whereas patients with OHS exhibited higher ESS scores and a greater prevalence of excessive DS.

**Conclusion:**

In patients with OSA, DS is not related to body weight or obesity per se. Instead, disease severity and the depth of nocturnal hypoxaemic events were the main determinants of excessive sleepiness, particularly in patients with concomitant OHS.

**Supplementary Information:**

The online version contains supplementary material available at 10.1007/s11325-026-03658-3.

## Introduction

Obstructive sleep apnoea (OSA) is characterised by recurrent sleep-related airflow interruption, leading to apnoeic events and resulting in intermittent hypoxaemia and sleep fragmentation [1]. In patients with OSA, quantitative measures of apnoea-related hypoxic burden [2], severity of sleepiness [3], cardiac autonomic response, and respiratory arousal intensity have emerged markers of increased cardiovascular risk [4]. OSA commonly occurs in individuals with obesity [5] and may contribute to cardiometabolic risk by exerting detrimental effects on adipose tissue metabolism and potentiating the adipose tissue dysfunction of obese subjects [6]. Unlike OSA, obesity hypoventilation syndrome (OHS) is characterised by the coexistence of obesity, defined by a body mass index (BMI) ≥ 30 kg/m^2^, daytime hypercapnia with an arterial partial pressure of carbon dioxide (PaCO_2_) ≥ 45 mmHg, and sleep-disordered breathing, resulting in alveolar hypoventilation [7]. Approximately 90% of patients with OHS also have OSA [7].

Daytime sleepiness (DS) represents a public health concern with heterogeneous aetiologies, generally related to sleep deprivation, sleep fragmentation, or sleep disorders [8]. Patients with moderate to severe OSA may be predisposed to perceive excessive DS [9]. Sleep irregularity and circadian disruption may represent underlying mechanisms linking subtypes of excessive DS to an increased risk of cardiovascular diseases [10]. Chronic intermittent hypoxia and sleep fragmentation, leading to oxidative injury and neuronal and brain network alterations involving noradrenergic and dopaminergic neurotransmission in wake-promoting brain regions, have been proposed as pathophysiological mechanisms underlying excessive DS in patients with OSA treated with Continuous Positive Airway Pressure (CPAP) [11]. The Epworth Sleepiness Scale (ESS) is a widely used questionnaire for the assessment of DS [12]. ESS scores have demonstrated robust test-retest reliability in evaluating treatment response over time across three large clinical trials involving patients with OSA [13]. In an obesity-matched case-control study, patients with OHS exhibited higher ESS scores than patients with OSA alone [14].

A causal relationship between body weight and DS have been reported. Among bus drivers, overweight and obesity are associated with an increased adjusted risk of excessive DS by nearly threefold and fourfold, respectively [15]. Short sleep duration and excessive DS are associated with greater odds of central obesity, independent of diet and physical activity [16]. Weight gain is associated with DS, as assessed by the ESS, with approximately one-fifth of this relationship attributed to OSA and approximately one-sixth to poor overall physical health [17]. Conversely, weight-loss interventions, including both surgical [18] and non-surgical approaches [18, 19], result in significant improvements in DS. Despite this evidence, no studies have specifically evaluated the relationship between body weight and DS in the context of sleep-disordered breathing, distinguishing between patients with OSA alone and those with combined OSA and OHS, all in the absence of treatment. We hypothesised that the burden of sleep-disordered breathing (OSA or OHS), characterised by chronic hypoxia and sleep fragmentation, may influence DS perception more strongly than body weight or obesity per se. Specifically, in untreated patients initially diagnosed with OSA, we hypothesised that body weight would not be closely associated with DS. Therefore, we aimed to evaluate the relationship between body weight and DS in patients with OSA, considering untreated OSA with OHS as a separate category due to the higher DS perception reported in these patients [14]. Finally, we sought to identify factors associated with excessive DS perception (ESS > 10) [12] and to explore any potential relationship with body weight categories.

## Methods

### Study cohort and groups

Between September 2020 and July 2024, we prospectively evaluated subjects referred for a suspected nocturnal respiratory disorder to the dedicated outpatient clinic of the Respiratory Medicine Unit at the Azienda Ospedaliera Universitaria Integrata of Verona. We excluded: (1) subjects with normal polygraphy; (2) patients with OSA or OHS receiving any form treatment; (3) patients with causes of nocturnal hypoventilation other than OHS [20]; and (4) patients with missing or of poor quality respiratory polygraphy data.

Based on body weight, patients with OSA were categorised as normal weight (BMI 18.5–24.9 kg/m^2^), overweight (BMI 25.0–29.9 kg/m^2^), grade 1 obesity (BMI 30.0–34.9 kg/m^2^), grade 2 obesity (BMI 35.0–39.9 kg/m^2^), grade 3 obesity (BMI ≥ 40.0) [21, 22], and OHS [7].

The study protocol was approved by the hospital ethics committee and conducted in accordance with Good Clinical Practices guidelines and the Declaration of Helsinki.

### General measures

Demographic and anthropometric characteristics, including neck, waist, and hip circumferences, prevalence of arterial hypertension, heart disease, diabetes, and peripheral arterial disease (PAD), as well as lifestyle information (smoking habits and pack-years), were collected.

### Nocturnal polygraphy and daytime sleepiness

Respiratory polygraphy was performed using a portable Nox T3s™ device (https://noxmedical.com, USA). Only recordings with adequate signal quality were included. The following signals were acquired: peripheral oxygen saturation (SpO_2_) measured by a finger sensor, thoracic and abdominal movements assessed by inductive belts, nasal airflow derived from the belts, snoring, and body position. This portable device has demonstrated excellent measurement agreement with to in-lab polysomnography [23]. The device’s signals were analysed using the Noxturnal™ software, with an advanced automated respiratory scoring algorithm. Each recording was subsequently reviewed by a pulmonologist with expertise in sleep medicine, who confirmed respiratory events and the final scoring. According to the American Academy of Sleep Medicine International Classification of Sleep Disorders, third edition [24], respiratory polygraphy includes total sleep time (TST) and the apnoea-hypopnoea index (AHI), calculated as the number of apnoeas and hypopnoeas per hour of sleep (n/h). Apnoea was defined as the absence of airflow for ≥ 10 s, while a hypopnoea was defined as a ≥ 30% reduction in airflow associated with an oxygen desaturation ≥ 3%. AHI severity was classified as mild (AHI ≥ 5 and ≤ 15 events/h), moderate (AHI > 15 and ≤ 30 events/h), and severe (AHI > 30 events/h) [1]. OHS was defined according to established criteria [7].

Pulse oximetry parameters included lowest SpO_2_, oxygen desaturation index (ODI, defined as the number of events per hour of oxygen desaturation of 3% ODI), mean desaturation depth, and sleep time with SpO_2_ < 90% (ST_90_) [1].

Daytime sleepiness was assessed using the Italian version of the ESS [25], an eight-items self-reported questionnaire evaluating the likelihood of falling asleep in daily situations.Total scores range from 0 to 24 [12, 25], with ESS > 10 indicating excessive DS [12].

### Statistical analysis

The assumption of normality was assessed using the preliminary Kolmogorov-Smirnov test. Categorical variables are reported as number and percentage, and continuous variables as median with interquartile range (IQR). Group comparisons were performed using χ2 or Fisher’s exact tests for categorical variables and nonparametric Mann-Whitney U or Kruskal-Wallis H tests for continuous variables.

Associations between variables were evaluated using the Spearman’s correlation coefficient (ρ).

Univariate and multivariate mixed models were used to identify factors associated with excessive sleepiness perception (dependent variable: ESS > 10) [12]. The following variables were included in the analysis: age, sex, obesity grade (reference: normal weight), smoking status, neck, waist, and hip circumferences, arterial hypertension, heart disease, diabetes, PAD, AHI severity, ODI, lowest SpO_2_, mean desaturation, and ST_90_. Collinearity was assessed using Spearman correlations, and variables with strong correlations (r > |±0.50|) were excluded from multivariate models. Multicollinearity was evalated using the variance inflation factor (VIF). Odds ratios (ORs) and 95% confidence intervals (CIs) were calculated [26]. Model calibration was assessed using the Hosmer-Lemeshow goodness-of-fit test [27]. Receiver operating characteristic (ROC) analysis and area under the curve (AUC) were used to identify the cut-off values for continuous variables significantly associated with excessive DS in multivariate models (means desaturation).

All analyses were performed using IBM SPSS, version 17.0 (IBM Corp., Armonk, NY, USA), and p-value < 0.05 were considered statistically significant.

## Results

**Fig. 1:**
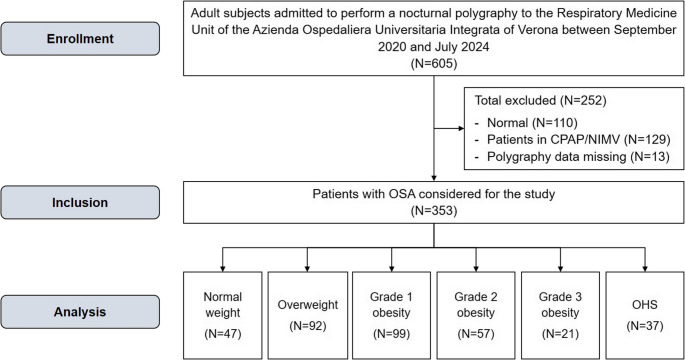
The study flow chart

A total of 353 OSA patients were included and categorised as normal weight (*N* = 47, 13%), overweight (*N* = 92, 26%), grade 1 obesity (*N* = 99, 28%), grade 2 obesity (*N* = 57, 16%), grade 3 obesity (*N* = 21, 6%), and OHS (*N* = 37, 11%). The study flowchart is reported in Fig. 1.

Significant differences among groups were observed in body circumferences, although values were comparable between patients with grade 3 obesity and those with OHS. Patients with OSA and grade 2 obesity showed a higher prevalence of arterial hypertension, heart disease and diabetes. Baseline clinical and anthropometric characteristics are summarised in Table 1.

**Table 1:** General characteristics of patients with OSA by body weight category

Variables	Normal weight (*N* = 47)	Overweight (*N* = 92)	Grade 1 obesity (*N* = 99)	Grade 2 obesity (*N* = 57)	Grade 3 obesity (*N* = 21)	OHS (*N* = 37)	*p*-value
Age, years	59 [18]	67 [15]^*^	66 [20]^*^	63 [22]	56 [14]^§ #^	58 [16]^§§ #^^	**< 0.001**
Male, n (%)	30 (64)	73 (79)	80 (81)	44 (77)	12 (57)	30 (81)	0.066
BMI, kg/m^2^	23.3 [1.6]	27.6 [1.8]^**^	32.5 [2.4]^**§§^	37.5 [2.5]^**§§##^	45.7 [7.5]^**§§##^^^	43.9 [7.4]^**§§##^^^	**< 0.001**
Circumferences, cm							
Neck	38 [6]	41 [3]^**^	42 [4]^**§§^	44 [3.8]^** §§##^	47 [8]^**§§##^	47.5 [5]^**§§##^^^	**< 0.001**
Waist	91 [11]	102 [7]^**^	114 [11]^**§§^	123 [8.8]^**§§##^	135 [18.5]^**§§##^^^	134.5 [15.8]^**§§##^^^	**< 0.001**
Hip	100 [9]	106 [5]^**^	113 [7]^**§§^	122 [8.8]^**§§##^	136 [13.5]^**§§##^^^	131 [16]^**§§##^^^	**< 0.001**
Smoking habit, n (%)							
Former/current smokers	16 (36)/7 (16)	49 (55)/12 (13)	54 (56)/11 (11)	24 (43)/13 (23)	7 (35)/3 (15)	15 (43)/5 (14)	0.284
Arterial hypertension, n (%)	15 (32)	44 (48)	49 (49)^*^	39 (68)^**§#^	12 (57)^*^	23 (62)^*^	**0.006**
Heart disease, n (%)	7 (15)	29 (31)^*^	29 (29)	27 (47)^**#^	6 (29)	9 (24)^^^	**0.016**
Diabetes, n (%)	4 (8.5)	9 (9.8)	19 (19)	22 (39)^**§§#^	6 (29)^*§^	9 (24)^*§^	**< 0.001**
PAD, n (%)	1 (2.1)	6 (6.5)	5 (5.1)	8 (14)	4 (19)	4 (11)	0.064

The data are reported as the number of patients (percentage) or medians [interquartile range]. Percentages are calculated for non-missing data. In bold are reported significant values

^*^ and ^**^ represent *p* < 0.05 and *p* < 0.001 versus normal weight; ^§^ and ^§§^ represent *p* < 0.05 and *p* < 0.001 versus overweight; ^#^ and ^##^ represent *p* < 0.05 and *p* < 0.001 versus grade 1 obesity; ^^^ and ^^^^ represent *p* < 0.05 and *p* < 0.001 versus grade 2 obesity; ^&^ and ^&&^ represent *p* < 0.05 and *p* < 0.001 versus grade 3 obesity

Nocturnal polygraphy data are reported in Table 2. Marked differences were observed in the OHS group, which was characterised by higher AHI values, a greater proportion of patients with severe OSA, higher ODI, greater mean desaturation depth and ST_90_, and lower lowest SpO_2_ values. No significant differences were observed in total sleep time spent in the supine position across groups.

**Table 2:** Nocturnal polygraphy data

Variables	Normal weight (*N* = 47)	Overweight (*N* = 92)	Grade 1 obesity (*N* = 99)	Grade 2 obesity (*N* = 57)	Grade 3 obesity (*N* = 21)	OHS (*N* = 37)	*p*-value
TST, min	463 [32]	461 [34]	460 [50]	456 [36]	464 [35]	460 [90.5]	0.889
Supine position of TST, %	47 [55]	36 [38]	34 [42]	36 [45]	37 [56]	36 [57]	0.545
AHI, n/h	11.4 [13.1]	18 [17.5]^*^	16.2 [20.4]	20.4 [25.8]^*#^	22.4 [21.7]^*^	40.8 [30.5]^**§§##^^&^	**< 0.001**
AHI stages, n (%)							**< 0.001**
Mild	32 (68)	41 (44)	48 (49)	19 (33)	6 (29)	3 (8)	
Moderate	6 (13)	31 (34)	28 (28)	17 (30)	8 (38)	8 (22)	
Severe	9 (19)	20 (22)	23 (23)	21 (37)	7 (33)	26 (70)	
ODI, n/h	12.6 [13.7]	21.1 [18.5]^*^	21.5 [21.2]^*^	27.2 [33.4]^**§^	28.6 [24.2]^**^	56.4 [39.6]^**§§##^^&^	**< 0.001**
Lowest SpO_2_, %	81 [10]	80 [7.8]	79 [8]	74 [13.5]^**§§#^	81 [10.5]^^^	63 [14]^**§§##^^&&^	**< 0.001**
Mean desaturation, %	4.8 [2.2]	5 [2]	4.9 [2.1]	6.3 [4]^*§#^	4.9 [3.1]	8.1 [5.9]^**§§##^&&^	**< 0.001**
ST_90_, %	3.5 [12.2]	5.5 [16.3]	10.7 [30.1]^*§^	21.8 [36.6]^**§§^	8.3 [12.4]^^^	57.6 [28.6]^**§§##^^&&^	**< 0.001**

The data are reported as the number of patients (percentage), mean ± standard deviation or medians [interquartile range]. In bold are reported significant values

^*^ and ^**^ represent *p* < 0.05 and *p* < 0.001 versus normal weight; ^§^ and ^§§^ represent *p* < 0.05 and *p* < 0.001 versus overweight; ^#^ and ^##^ represent *p* < 0.05 and *p* < 0.001 versus grade 1 obesity; ^^^ and ^^^^ represent *p* < 0.05 and *p* < 0.001 versus grade 2 obesity; ^&^ and ^&&^ represent *p* < 0.05 and *p* < 0.001 versus grade 3 obesity

**Fig. 2:**
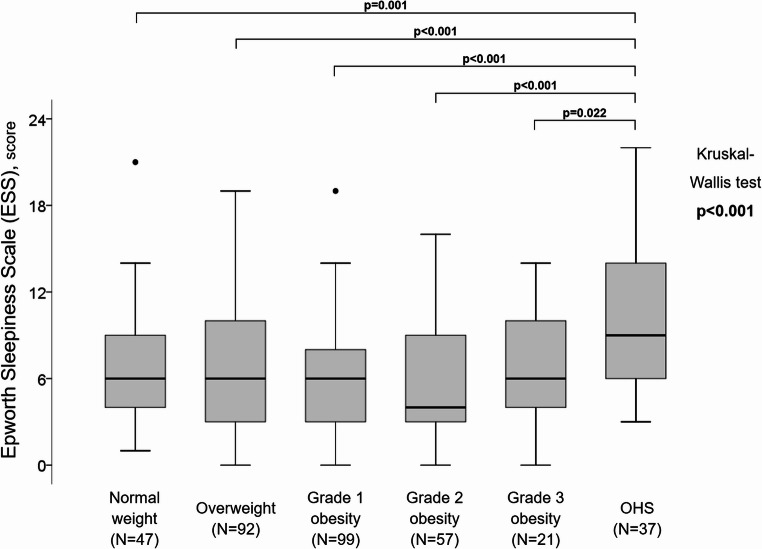
Boxplot of daytime sleepiness by study groups

DS scores were significantly higher in patients with OHS compared with all other groups (Kruskal-Wallis test, *p* < 0.001) (Fig. 2). Consistently, the prevalence of excessive DS perception was also higher in the OHS group (Supplementary Fig. 1).

Correlation analyses are reported in Table 3. ESS scores showed strong correlations with age, AHI, ODI, lowest SpO_2_, and mean desaturation depth (all *p* < 0.001). Weaker but significant correlations were observed with sex (*p* = 0.030), neck circumference (*p* = 0.016), and ST_90_ (*p* = 0.037). No significant correlation was found between ESS and BMI.

**Table 3:** Correlation analyses

	Variables	ESS	2	3	4	5	6	7	8	9	10
2	Age	-ρ -0.228(*p* < 0.001)		-	-	-	-	-	-	-	-
3	Gender	ρ -0.118 (*p* = 0.030)	ρ 0.130 (*p* = 0.015)	-	-	-	-	-	-	-	-
4	BMI	ρ 0.083 (*p* = 0.126)	ρ -0.134 (*p* = 0.012)	ρ -0.016 (*p* = 0.758)	-	-	-	-	-	-	-
5	Circumference neck	ρ 0.131 (*p* = 0.016)	ρ -0.136 (*p* = 0.012)	ρ -0.494 (*p* < 0.001)	ρ 0.669 (*p* < 0.001)	-	-	-	-	-	-
6	AHI	ρ 0.232 (*p* < 0.001)	ρ -0.065 (*p* = 0.226)	ρ -0.167 (*p* = 0.002)	ρ 0.288 (*p* < 0.001)	ρ 0.319 (*p* < 0.001)	-	-	-	-	-
7	ODI	ρ 0.202 (*p* < 0.001)	ρ -0.055 (*p* = 0.299)	ρ -0.101 (*p* = 0.058)	ρ 0.363 (*p* < 0.001)	ρ 0.324 (*p* < 0.001)	ρ 0.885 (*p* < 0.001)	-	-	-	-
8	Lowest SpO_2_	ρ -0.187 (*p* < 0.001)	ρ -0.011 (*p* = 0.832)	ρ -0.030 (*p* = 0.579)	ρ -0.341 (*p* < 0.001)	ρ -0.261 (*p* < 0.001)	ρ -0.420 (*p* < 0.001)	ρ -0.422 (*p* < 0.001)	-	-	-
9	Mean desaturation	ρ 0.255 (*p* < 0.001)	ρ 0.011 (*p* = 0.838)	ρ -0.065 (*p* = 0.226)	ρ 0.282 (*p* < 0.001)	ρ 0.221 (*p* < 0.001)	ρ 0.540 (*p* < 0.001)	ρ 0.480 (*p* < 0.001)	ρ -0.706 (*p* < 0.001)	-	-
10	ST_90_	ρ 0.113 (*p* = 0.037)	ρ 0.074 (*p* = 0.168)	ρ 0.007 (*p* = 0.892)	ρ 0.399 (*p* < 0.001)	ρ 0.288 (*p* < 0.001)	ρ 0.426 (*p* < 0.001)	ρ 0.460 (*p* < 0.001)	ρ -0.705 (*p* < 0.001)	ρ 0.558 (*p* < 0.001)	-
11	Supine position	ρ 0.065 (*p* = 0.241)	ρ 0.011 (*p* = 0.844)	ρ 0.112 (*p* = 0.038)	ρ -0.017 (*p* = 0.750)	ρ -0.082 (*p* = 0.137)	ρ 0.126 (*p* = 0.019)	ρ 0.147 (*p* = 0.006)	ρ -0.108 (*p* = 0.045)	ρ 0.156 (*p* = 0.004)	ρ 0.045 (*p* = 0.412)

Bold text indicates a statistically significant difference. Abbreviations: *ESS* indicates Epworth Sleepiness Scale, *BMI* body mass index, *AHI* apnoea-hypopnoea index, *ODI* oxygen desaturation index, *SpO2* pulse oximetry oxygen saturation, *ST90* sleep time with SpO2 below 90%.

Univariate logistic regression analysis (Table 4) showed that patients with OHS had a significantly increased risk of excessive DS perception (ESS > 10) compared with normal-weight patients with OSA (OR 3.87; *p* = 0.011). Moderate and severe OSA were also associated with excessive DS compared with mild OSA (OR 3.04; *p* = 0.003 and OR 4.48; *p* < 0.001, respectively). Excessive DS was directly associated with ODI (OR 1.019; *p* = 0.001), mean desaturation depth (OR 1.24; *p* < 0.001) and ST_90_ (OR 1.011; *p* = 0.033), and inversely associated with lowest SpO_2_ (OR 0.94; *p* < 0.001). In the multivariate analysis, AHI severity (moderate and severe stages) and mean desaturation depth remained independently associated with excessive DS perception.

**Table 4:** Mixed model effects assessing factors associated with excessive sleepiness perception

Depending variable: ESS > 10 (*N* = 67)	Univariate			Multivariate		
Variables	OR	95% CI	p-value	OR	95% CI	p-value
Body weight						
Normal weight	1	-	-			
Overweight	1.47	0.57 to 3.82	0.425			
Grade 1 obesity	0.68	0.25 to 1.91	0.470			
Grade 2 obesity	1.21	0.42 to 3.47	0.728			
Grade 3 obesity	1.81	0.49 to 6.60	0.369			
OHS	3.87	1.37 to 11.0	**0.011**			
AHI stages						
Mild	1	-	-	1	-	-
Moderate	3.04	1.44 to 6.38	**0.003**	2.52	1.18 to 5.38	**0.017**
Severe	4.48	2.21 to 9.07	**< 0.001**	2.47	1.11 to 5.50	**0.026**
ODI, n/h	1.019	1.008 to 1.030	**0.001**			
Lowest SpO_2_, %	0.94	0.92 to 0.97	**< 0.001**			
Mean desaturation, %	1.24	1.14 to 1.36	**< 0.001**	1.18	1.07 to 1.31	**0.001**
ST_90_, %	1.011	1.001 to 1.020	**0.033**			

Hosmer-Lemeshow test *p* = 0.765. In bold are reported significant values

Abbreviations: *ESS* indicates Epworth Sleepiness Scale, *OR* odds ratio, *CI* confidence interval, *OHS* obesity hypoventilation syndrome, *AHI*apnoea-hypopnoea index, *ODI* oxygen desaturation index, *SpO*_*2*_ pulse oximetry oxygen saturation, *ST*_*90*_ sleep time with SpO_2_ below 90%

**Fig. 3:**
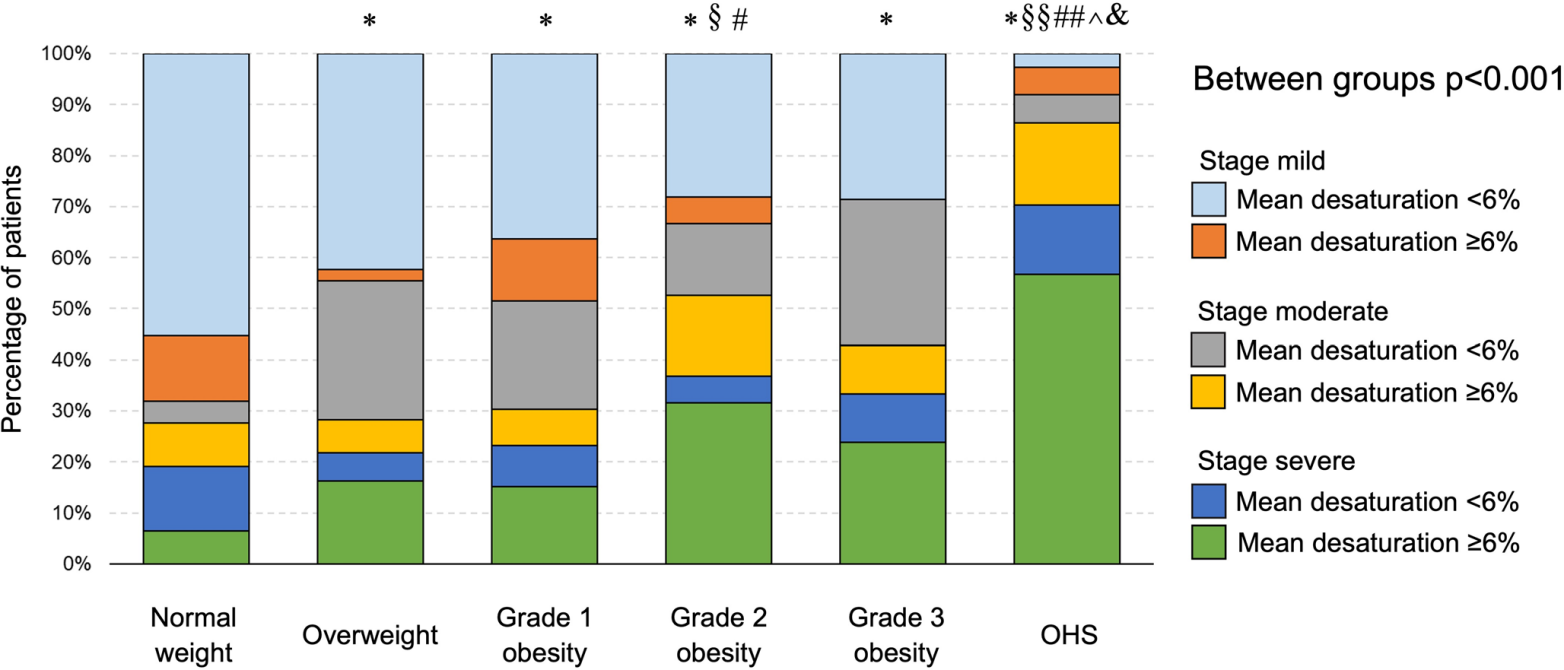
Distribution of patients, stratified by OSA severity and the mean desaturation cut-off value

ROC curve analysis identified a mean desaturation cut-off value of 5.65% (AUC 0.664; *p* < 0.001), which was approximated to 6% for clinical applicability (Supplementary Fig. 2). The regression model incorporating AHI severity and mean desaturation categories (Table 5) showed significant associations with excessive DS for moderate OSA with mean desaturation ≥ 6% (OR 5.94; *p* < 0.001), and for severe OSA with mean desaturation < 6% (OR 3.15; *p* = 0.032) and ≥ 6% (OR 4.95; *p* < 0.001). These three categories of patients, all associated with excessive DS perception, were predominantly represented among patients with OHS (87%), compared with normal weight (28%), overweight (28%), grade 1 obesity (30%), grade 2 obesity (53%), and grade 3 obesity (43%) (*p* < 0.001 between groups) (Fig. 3).

**Table 5:** Logistic regression analyses using categorical variables related to OSA severity and mean desaturation to assess factors associated with perceived excessive sleepiness (ESS > 10; *N* = 67)

Variables	OR	95% CI	*p*-value
Stage mild			
Mean desaturation < 6% (*N* = 124)	1	-	-
Mean desaturation ≥ 6% (*N* = 25)	0.90	0.19 to 4.35	0.897
Stage moderate			
Mean desaturation < 6% (*N* = 64)	1.87	0.75 to 4.68	0.181
Mean desaturation ≥ 6% (*N* = 34)	5.94	2.31 to 15.3	**< 0.001**
Stage severe			
Mean desaturation < 6% (*N* = 29)	3.15	1.10 to 9.03	**0.032**
Mean desaturation ≥ 6% (*N* = 77)	4.95	2.26 to 10.8	**< 0.001**

Bold text indicates a statistically significant difference.0.8970.181<0.0010.032<0.001Abbreviations: *ESS* indicates Epworth Sleepiness Scale, *OR* odds ratio, *CI* confidence interval, *OSA* obstructive sleep apnea

## Discussion

Multiple lines of evidence support an association between obesity and DS [15–19]. However, in patients with OSA - who are generally obese [5, 6] and whose excessive DS [8] is driven by a combination of central and peripheral mechanisms related to sleep-disordered breathing [11] - this relationship may be less direct, with nocturnal respiratory disease exerting a stronger influence on sleepiness than body weight alone. Our findings demonstrate that untreated patients with OSA across a wide range of body weight categories, including different grades of obesity, exhibit comparable levels of DS, with no clear association between body weight and ESS scores. The notable exception is represented by patients with OSA and concomitant OHS, who display significantly greater DS perception. Disease severity, reflected by AHI stage and the depth of nocturnal hypoxaemic events (mean desaturation), appears to be a key determinant of symptom perception.

DS is recognised as a marker of disease severity in OSA, particularly in relation to cardiovascular risk [3]. Even in patients treated with CPAP, persistent excessive DS has been linked to neuronal and brain network alterations [11]. For the first time, our study stratifies untreated patients with OSA according to body weight categories, allowing a detailed evaluation of DS perception across distinct phenotypes with different baseline and nocturnal characteristics (Tables 1 and 2). This approach extends beyond prior case-control studies comparing OSA and OHS, in which all participants were obese [14]. In the referenced study [14], patients with OHS exhibited significantly worse nocturnal oxygenation (lowest SpO_2_ and ST_90_) but did not differ from OSA patients in terms of AHI or ODI. In contrast, our patients with OSA and OHS showed significantly higher AHI values, confirming the greatest severity of sleep-disordered breathing in this condition [7]. This findings is consistent with evidence indicating that patients with OHS experience longer obstructive apnoeas and hypopnoeas than those with OSA alone, reflecting an impaired ventilatory compensation [20, 28]. Although body posture may influence sleep quality in CPAP-treated patients with OSA [29], supine sleep time did not differ among groups in our untreated cohort and was not associated with DS perception.

The absence of a relationship between body weight and DS - except in patients with OHS - is supported by the lack of differences in ESS distribution across weight categories (Fig. 2) and by the absence of a significant correlation between BMI and ESS (ρ = 0.083, *p* = 0.126) (Table 3). Our patients with OSA and OHS were characterised not only by obesity (at least grade 1) but also daytime hypercapnia and alveolar hypoventilation, resulting in a distinct nocturnal respiratory profile [7]. These patients exhibited significantly worse indices of nocturnal intermittent and severe hypoxia, including ODI, lowest SpO_2_, mean desaturation depth, and ST_90_ (Table 2). In this context, the greater DS perception observed in patients with OSA and OHS (Figs. 2 and Supplementary Fig. 1) is likely driven by the severity of nocturnal hypoxaemia. Consistent with previous studies [30, 31], hypoxic parameters were strongly associated with an increased risk of excessive DS (ESS > 10) (Table 4). The combination of higher AHI severity and greater mean desaturation - categorised using the ROC-derived cut-off of 6% (Supplementary Fig. 2) - allowed us to identify patient subgroups at particularly high risk of excessive DS, most of whom belonged to the OHS group (Fig. 3; Table 5). Conceptually, our patients with OSA and OHS, irrespective of obesity severity, may represent a subgroup with a worse cardiovascular prognosis, given the close association between severe nocturnal respiratory disturbances and pronounced DS perception, both of which are predictors of adverse cardiovascular outcomes [2, 3, 32]. Notably, all findings reported here pertain to untreated patients, underscoring the dominant role of nocturnal abnormalities in driving DS. Effective treatment of sleep-disordered breathing may therefore help disentangle the contribution of body weight from that of respiratory disease in the perception of sleepiness.

A key strength of this study is the novel stratification of patients with OSA according to body weight, which enables a more nuanced interpretation of DS perception as a symptom primarily related to sleep-disordered breathing rather than obesity itself. Consequently, ESS assessment should also be routinely performed in normal-weight patients with OSA to accurately capture symptom burden.

Several limitations should be acknowledged. The relatively small sample size within each subgroup reflects the case mix of a single dedicated centre and may limit generalisability. Nevertheless, the distribution of body weight categories in our cohort is consistent with epidemiological data on OSA [1, 5, 6], with more than half of patients clssfied as obese and over one- quarter as overweight. The prevalence of OHS (approximately 10%) aligns with previous reports [20, 33]. BMI remains the most commonly used anthropometric index in clinical practice due to its simplicity, but it does not provide information on body composition or fat distribution [34]. In particular, BMI does not distinguish between lean and fat mass or between visceral and subcutaneous adiposity, which have distinct metabolic and cardiovascular implications [35]. Further studies incorporating body composition assessment are warranted to refine obesity phenotyping [36]. Moreover, nocturnal polygraphy was used instead of in-lab polysomnography. Although unlikely to have influenced the main findings, polysomnography could have provided additional information on arousal-related events. Finally, while the ESS may offer functional advantages over objectives tests such as the multiple sleep latency test (MSLT) in OSA [37], subjective sleepiness perception may not always reflect objective sleep disruption, potentially leading to undeestinmation of disease severity [38].

## Conclusion

In untreated patients with OSA, body weight - including obesity - is no associated with daytime sleepiness. Instead, the number of apnoeic events and the depth of nocturnal hypoxaemic episodes (mean desaturation) appear to be the primary determinant of excessive sleepiness, particularly in patients with concomitant OHS. 

## Supplementary Information

Below is the link to the electronic supplementary material.

Supplementary Material 1

Supplementary Material 2

## Data Availability

The datasets used and/or analysed during the current study are available from the corresponding author on reasonable request.

## Declarations

### linical trial registration

This study was not registered.
