# Supplementary figures and images for: Relationship between body weight and daytime sleepiness in patients with untreated OSA: The role of hypoventilation due to obesity
Source: Sleep Breath. 2026 Mar 22;30(2):103. doi: 10.1007/s11325-026-03658-3 (PMC13005857; doi:10.1007/s11325-026-03658-3)

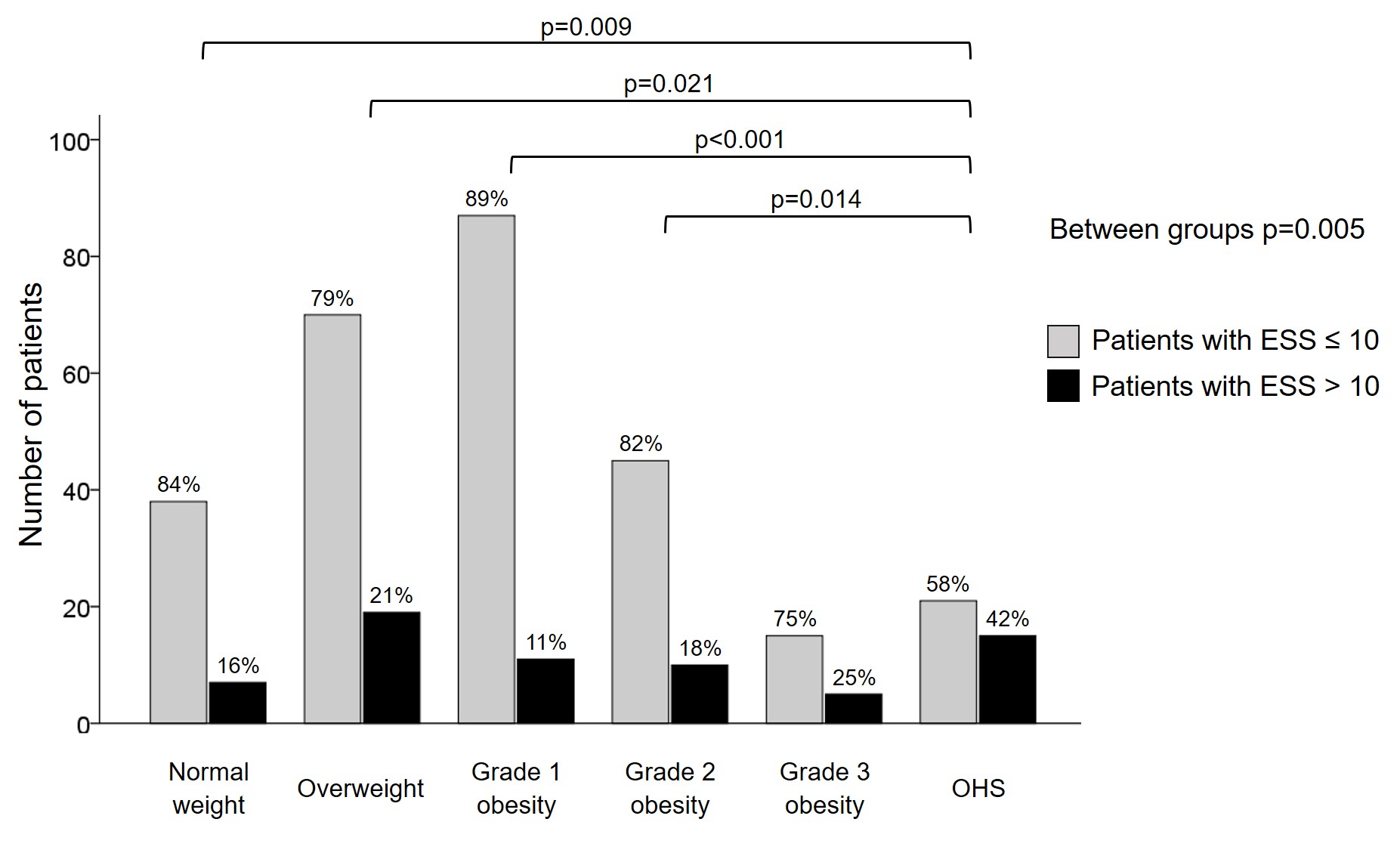

Supplement: Supplementary file 1 — Supplementary Material 1 [file 11325_2026_3658_MOESM1_ESM.jpg]

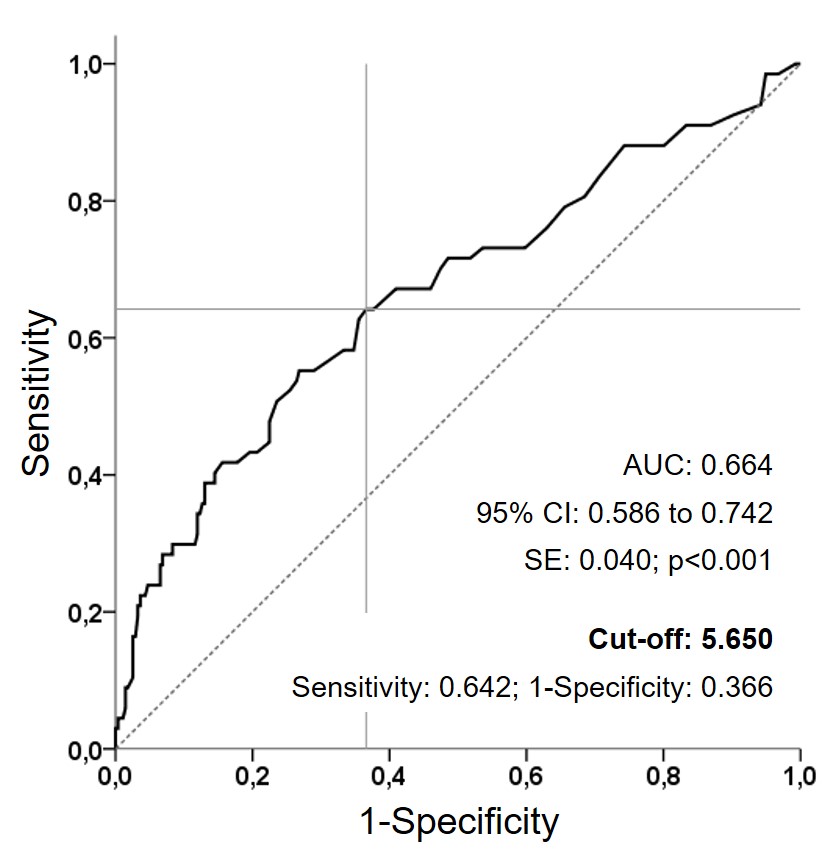

Supplement: Supplementary file 2 — Supplementary Material 2 [file 11325_2026_3658_MOESM2_ESM.jpg]
